# Joint exploration of network pharmacology and metabolomics on the effects of traditional Chinese medicine compounds in weaned yaks

**DOI:** 10.3389/fvets.2024.1511311

**Published:** 2025-01-13

**Authors:** Sijia Lu, Yanbin Zhu, Xiaojuan Zhang, Yangji Cidan, Wangdui Basang, Kun Li

**Affiliations:** ^1^College of Veterinary Medicine, Nanjing Agricultural University, Nanjing, China; ^2^Institute of Animal Husbandry and Veterinary Medicine, Tibet Academy of Agriculture and Animal Husbandry Sciences, Lhasa, China; ^3^College of Animal Science and Technology, Gansu Agricultural University, Lanzhou, China; ^4^Agriculture and Animal Husbandry Science and Technology Service Station in Seni District, Naqu, China

**Keywords:** traditional Chinese medicine compounds, weaned yaks, network pharmacology analysis, metabolomics, diarrhea

## Abstract

**Introduction:**

Chinese herbal medicines are relatively inexpensive and have fewer side effects, making them an effective option for improving health and treating diseases. As a result, they have gained more attention in recent years. The weaning period is a critical stage in the life of yaks, often inducing stress in calves. Weaning stress, along with dietary changes, can lead to a decline in physical fitness and immune function, making yaks more susceptible to diarrhea and resulting in high mortality rates during this period. Therefore, our study aimed to address this issue by incorporating traditional Chinese medicine (TCM) formulas into the diet of yaks during the weaning period.

**Methods:**

Following a dialectical analysis, three TCM formulas, mainly composed of *Paeonia lactiflora, Coptis chinensis*, and *Dandelion*, were identified for their anti-inflammatory, antioxidant, and immune enhancing potentials. We explored the possible molecular mechanisms of these TCM formulas using network pharmacology analysis and investigated their effects on the physiology of yaks through metabolomics.

**Results:**

Network pharmacology analysis revealed several key target proteins in the protein–protein interaction (PPI) network between three formulas and immune-related genes, including *PIK3R1*, *PIK3CA*, *JAK2*, *PTK2,* and *PYPN11*. The key target proteins in the PPI network associated with metabolism-related genes included *ENPP1*, *CYP1A1*, *PTGS1*, members of the *CYP1* family, and *EPHX2*. GO analysis of co-targets revealed highly enriched pathways such as protein phosphorylation, plasma membrane, and one-carbon metabolic processes. Metabolomics revealed significant changes in the abundance of metabolites including dimethyl sulfoxide, tyrphostin A25, and thromboxane A2 in the intestines of weaned yaks supplemented with these Chinese herbal compounds. Significant changes were also observed in pathways such as vitamin A metabolism, chloroalkane, and chloroalkene degradation.

**Discussion:**

Based on these findings, it can be inferred that TCM formulas improve the physical fitness of weaned yaks by enhancing antioxidant capacity, boosting immunity, and reducing intestinal inflammation. This study preliminarily elucidates the pharmacological mechanisms by which TCM formulas prevent diarrhea and improve physical fitness in weaned yaks through metabolomics and network pharmacology, paving the way for further evaluation of the effectiveness of these three formulas.

## Introduction

Yaks are an ancient breed native to China, primarily distributed in high-altitude areas of the Qinghai Tibet Plateau and surrounding areas, including Gansu and Sichuan provinces (above 3,000 m). Yaks have been domesticated by nomadic tribes for over 7,300 years (1–3), providing essential survival resources such as fur, meat, and milk for local herders. The Qinghai Tibet Plateau is characterized by high altitude, low oxygen pressure, scarce resources, low temperatures, and extreme environmental conditions (3, 52). Yaks that have evolved in this environment possess unique physiological characteristics, allowing them to tolerate high-altitude climates, making them one of the main economic sources for the local people (4). However, yaks have slow growth rates, long pregnancy cycles, delayed weaning of calves, and high disease and mortality rates in calves, all of which significantly hinder the development of the local yak breeding industry (52). The weaning period presents a substantial challenge for yaks, during which they face social and psychological pressures such as malnutrition and separation from their mothers. These stressors lead to changes in behavior, metabolism, physiology, and immune status, increasing susceptibility to pathological conditions such as oxidative stress and diarrhea (5, 6).

Traditional Chinese herbal medicine (TCHM) is increasingly used to maintain health and prevent or treat diseases in both humans and animals due to its low toxicity, minimal side effects, and affordability. TCM is often administered as monotherapies or in compound formulas to reduce side effects and enhance therapeutic effects (7). In animal husbandry, TCHM formulas have been shown to improve the reproductive performance in cows during the perinatal period, antioxidant capacity (8), improve growth performance in weaned piglets (9), and alleviate heat stress in beef cattle, thereby increasing apparent nutrient digestibility (10).

In this study, *Coptis chinensis*, the root of Chinese *pulsatilla*, *Dandelion*, *Licorice,* and other herbs were selected as the main medicinal ingredients in the compound formula. *Coptis chinensis* has demonstrated significant therapeutic potential in treating metabolic and inflammatory diseases (11). Together with the root of Chinese *pulsatilla* and *Dandelion*, *Coptis chinensis* can alleviate intestinal inflammation, combat gastrointestinal diseases, and enhance antioxidant capacity (12–15). *Licorice*, a key herb in many formulas, helps harmonize the effects of other herbs and provides mild health benefits, making it widely used in TCHM (16).

This study adopted network pharmacology analysis to investigate the active ingredients in TCM used in the experiment, clarified their overall mechanism of action, and analyzed the principles of drug combination and prescription compatibility. The molecular correlation between drugs and therapeutic targets was explored to better understand the pharmacological effects of the compound formula. Metabolomics, an essential aspect of systems biology, allows for a comprehensive quantitative analysis of endogenous metabolites in real time, providing insight into the underlying mechanisms of drug action. When TCM interacts with the gut microbiota in the gastrointestinal tract, it can significantly alter the composition of gut microbiota metabolites (17). Preliminary experiments in this study demonstrated that Chinese herbal formulas reduced serum inflammatory markers, improved antioxidant capacity, and consequently enhanced the health and disease resistance of weaned yaks. Based on these findings, this study further investigates the rectal metabolism of weaned yaks using metabolomics to analyze and identify metabolites and metabolic pathways in rectal contents, exploring the effects of Chinese herbal compounds on weaned yaks. Conclusively, this study shows that TCM compounds can promote the health and growth of weaned yaks as an alternative to antibiotics, contributing to strategies to mitigate antibiotic resistance in the management of yaks.

## Materials and methods

### Experimental design and sample collection

The experiment was carried out at the Gesangtang Yak Breeding Base (Linzhou County, Lhasa, Xizang). Twenty-four newly weaned yaks (approximately 6 months old) were used and divided randomly into four groups. The treatment groups were as follows:

1. XA: 5% dietary addition of TCM formula I which consisted of 18.18% *Rhizoma coptidis*, 13.64% *Pulsatilla chinensis* (Bunge) Regel, 13.64% *Dark plum,* 13.64% *Myrobalan*, 13.64% *Xizang Rheum officinale*, 13.64% *Plantain Seed,* and 13.64% *Glycyrrhiza uralensis* Fisch.
2. XB: 5% dietary addition of TCM formula II, which consisted of 25% *Pulsatilla chinensis* (Bunge) Regel, 12.5% *Rhizoma Coptidis*, 12.5% *Artemisia Scoparia*, 12.5% *Tibet Inula Root*, 12.5% *White Peony*, 12.5% *Anisodamine*, and 12.5% *Glycyrrhiza uralensis* Fisch.
3. XC: 5% dietary addition of TCM formula III, which consisted of 30% *Taraxacum mongolicum*, 25% *Myrobalan*, 15% *Anisodamine*, 15% *Magnolia Officinalis*, and 15% *Pueraria Lobata*.
4. XD: Control group, fed *ad libitum*.

Randomization was employed to distribute yaks evenly across treatment groups, ensuring similar age, weight, and baseline health. Environmental factors, including feed type and housing conditions, were standardized to minimize confounding influences. The experimental period included 7 days of adaptation and 30 days of measurements. Samples were collected at two time points: on the 15th day (XZ1) and the 30th day after the start of the experiment (XZ2). Rectal content samples (3-5 g) were collected using disposable sterile swabs, which were inserted into the rectum. The samples were immediately placed in sterile fecal collection tubes and transported to the laboratory at low temperatures. The samples were stored at −80°C until analysis.

### Network pharmacology analysis

The Traditional Chinese Medicine System Pharmacological Analysis Platform (TCMSP) was used to screen the main components of TCM. Keywords such as “*Coptis chinensis,*” “*Chinese pulsatilla,*” “*Radix liquiritiae,*” and “*Taraxacum mongolicum*” were used to identify relevant ingredients. Oral bioavailability (≥ 30%) and drug likeness (≥ 0.18) were applied as filtering criteria in line with established pharmacokinetic screening methods to enhance the potential efficacy and bioactivity of the selected compounds in the yak model (18). The SMILES format of the main components was imported into the Swiss Target Prediction database to obtain the gene names for a major component. Target genes with a binding probability >0 were selected (19). Immune-related genes were retrieved from the IMMPORT database using the “Gene Lists” plugin, whereas metabolic-related genes were obtained from the KEGG database using R 4.3.3 (20). The active ingredients of TCM and their target genes were imported into Cytoscape 3.10.0 to construct an ingredient–target network.

Additionally, immune-related and metabolic-related genes were uploaded to the Bioinformatics Cloud Platform^1^ to generate Venn diagrams. Protein–protein interaction (PPI) networks were constructed using the STRING database (confidence score > 0.7), focusing on high-confidence interactions supported by experimental or computational evidence to minimize false positives. Visualization and network analysis were performed using Cytoscape [version 3.10.0 (21)]. Finally, the gene ontology (GO) functional and KEGG pathway enrichment analyses were performed using the DAVID database (22) and visual presentations were created using Micro Bioinformatics.^2^

### Untargeted metabolomics processing

The extraction of fecal metabolites and liquid chromatography–mass spectrometry (LC–MS) analysis were performed according to established methods (23, 24). Approximately 100 *μL* of each sample was put into 1.5-ml centrifuge tubes, and 400 *μL* 80% methanol was added and then vortexed and left in ice water for 5 min before centrifugation at 15,000 *g* for 20 min at 4 ͦ C. The supernatant was diluted to reach a methanol content of 53% and then centrifuged again under the same conditions. The final supernatant was injected into a high-resolution mass spectrometry system for analysis (25).

The UHPLC–MS/MS analysis was performed using a Vanquish UHPLC system (Thermo Fisher, Germany) with a Q Exactive™ HF mass spectrometer (Thermo Fisher, Germany). Chromatographic separation was achieved using a Hypersil Gold C18 column (100 × 2.1 mm, 1.9 μm) at 40°C with a 17-min linear gradient and a flow rate of 0.2 mL/min. Mobile phases for positive ion mode (ESI+) and negative ion mode (ESI–) were used (Supplementary Table 1). QC samples were injected into the system to monitor instrument stability and ensure accuracy (26). Quantification of metabolites was achieved through LC–MS analysis. Fold change (≥2.0 or ≤0.5) and *p*-values (< 0.05) were used to assess biological significance, while VIP scores (≥1.0) ensured the reliability of the identified changes.

### Data processing and identification of metabolites

The raw data files from UHPLC–MS/MS were processed using Compound Discoverer 3.1 (Thermo Fisher). Peak intensities were normalized to the total spectral intensity. Molecular formula predictions were made based on ion peaks matched against the mzCloud, mzVault, and MassList databases for identification. Metabolites with a coefficient of variation (CV) of less than 30% in the QC samples were retained for further analysis (27). Statistical analyses were conducted using R (version 3.4.3), Python (2.7.6 version), and CentOS (release 6.6).

### Bioinformatics and statistical analysis

Metabolite annotation was performed using the KEGG, HMDB,^3^ and LIPID MAPS databases.^4^ The multivariate statistical analysis, including principal component analysis(PCA) and orthogonal partial least squares discriminant analysis (OPLS-DA), was performed using metaX (54). Statistical significance (*p*-value) and fold change (FC) were calculated via *t*-test. One-way *ANOVA* was conducted using SPSS software (version 29.0, USA) for comparisons among different groups. Visualization tools R were used to generate clustering heatmaps, volcano plots, and bubble plots to identify significant metabolites. Hierarchical clustering and metabolite correlation analysis were employed to assess relationships between samples and metabolites. Statistical significance was set at *p*-value <0.05.

## Results

### Network pharmacology analysis

The main ingredients of the TCM in the three compound formulas are presented in Supplementary Tables 2–15. Supplementary Figure S1 shows the TCM ingredient–target spot network maps, which were constructed using Cytoscape 3.10.0 software.

**Figure 1:**
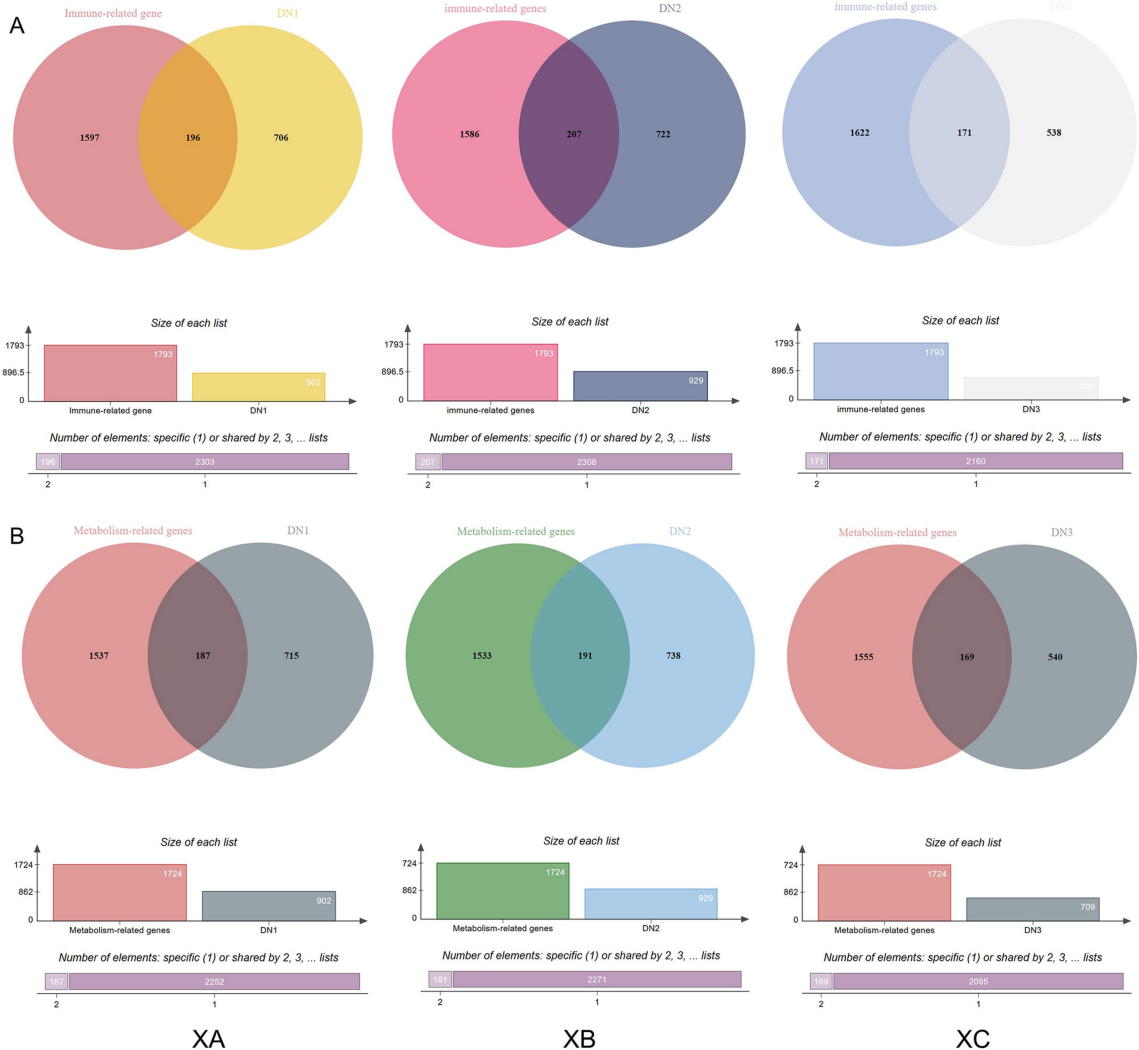
**(A)** Venn diagram of the interaction between the primary drug component genes and immune-related genes. **(B)** Venn diagram of the interaction between the primary drug component genes and metabolism-related genes. XA: Traditional Chinese medicine formula I. XB: Traditional Chinese medicine formula II. XC: Traditional Chinese medicine formula III.

A total of 1,793 immune-related genes were identified from traditional Chinese herbal medicine formula (TCHMF) 1, and 902 medicine ingredients target genes were obtained after deduplication. Venn diagrams were used to express the overlap between drug targets and immune-related genes, revealing 196 intersecting proteins between TCHMF1 and immune-related genes. Similarly, 1,793 immune-related genes were identified from TCHMF2, and after deduplication, 929 medicine ingredient target genes were obtained, showing 207 intersecting proteins between TCHMF2 and immune-related genes. For TCHMF3, 1,793 immune-related genes and 709 deduplicated medicine ingredient target genes were obtained, with 171 intersecting proteins identified between TCHMF3 and immune-related genes (Figure 1A). Additionally, 1732 metabolism-related genes were identified across the three traditional Chinese Herbal Medicine formulas. In TCHMF1, 902 medicine ingredient target genes were obtained after deduplication, and the Venn diagram revealed 187 intersecting proteins between TCHMF1 and metabolic-related genes. 929 deduplicated medicine ingredient target genes, with 191 intersecting proteins between TCHMF2 and metabolic-related genes. For TCHMF3, 709 deduplicated medicine ingredient target genes were identified with 69 intersecting proteins between TCHMF3 and metabolic-related genes (Figure 1B).

**Figure 2:**
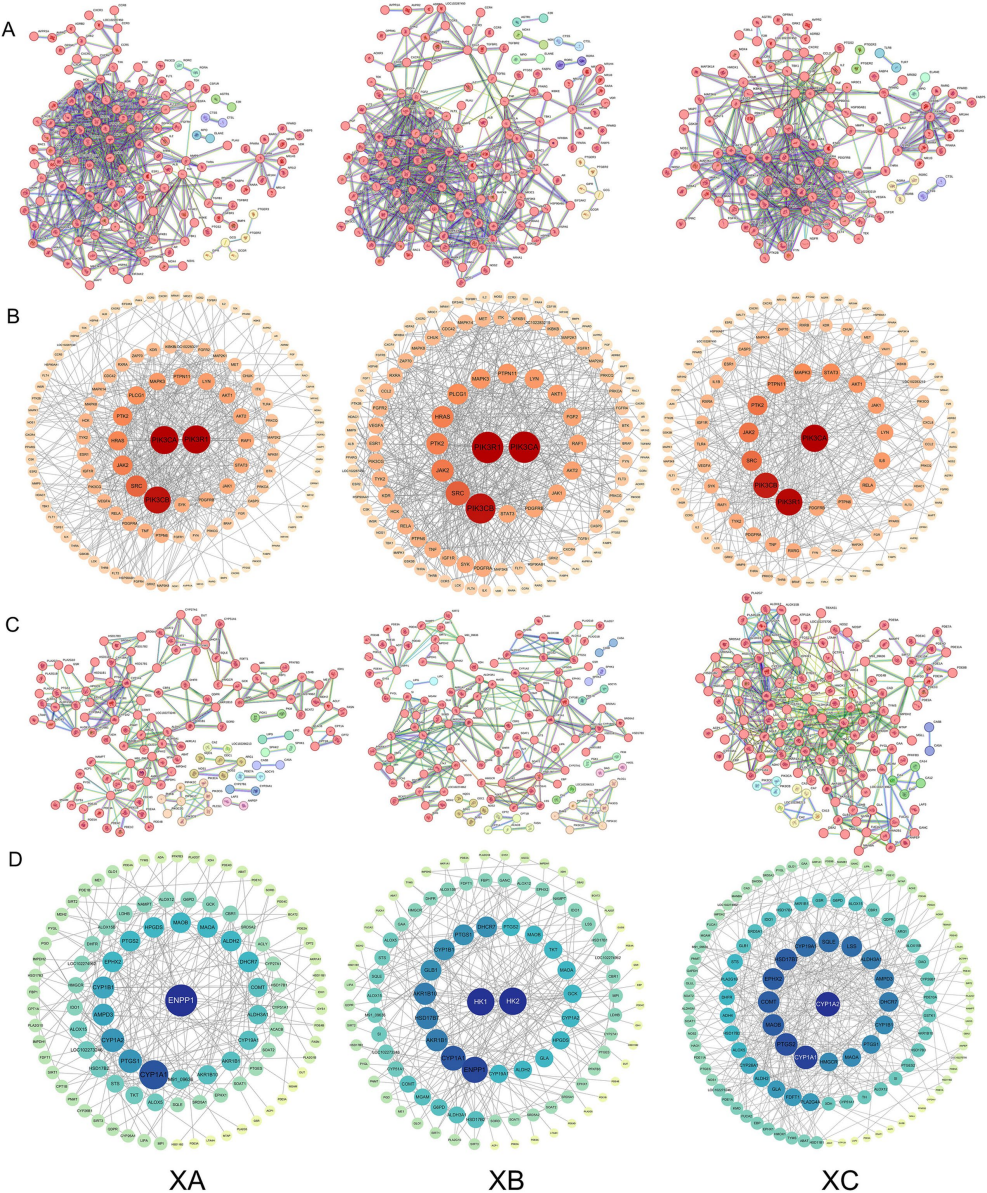
**(A)** Protein–protein interaction (PPI) network between traditional Chinese medicine formulas and immune-related genes. **(B)** Protein–protein interaction (PPI) network between traditional Chinese medicine formulas and immune-related genes (Cytoscape). **(C)** Protein–protein interaction (PPI) network between traditional Chinese medicine formulas and metabolism-related genes. **(D)** Protein–protein interaction (PPI) network visualization of traditional Chinese medicine formulas and metabolism-related genes (Cytoscape). XA: Traditional Chinese medicine formula I. XB: Traditional Chinese medicine formula II. XC: Traditional Chinese medicine formula III.

The protein–protein interaction (PPI) networks for the three compound formulas and immune/metabolism-related genes are illustrated in Figure 2. The size and color intensity of a node are directly proportional to its degree value; larger and darker nodes represent higher degree values. In the immune-related gene PPI network of TCHMF1, there were 179 nodes and 540 edges. The PPI network, constructed using Cytoscape 3.10.0, identified the top 10 core targets ranked by degree values: *PIK3R1*, *PIK3CA*, *PIK3CB*, *SRC*, *JAK2*, *HRAS*, *PTK2*, *PLCG1*, *MAPK3*, and *PTPN11*. The PPI network of TCHMF2 contained 189 nodes and 567 edges, with the top core targets being *PIK3R1*, *PIK3CA*, *PIK3CB*, *SRC*, *JAK2*, *PTK2*, *HRAS*, *PLCG1*, *MAPK3*, and *PTPN11*. For TCHMF3, the immune-related gene PPI network had 157 nodes and 435 edges, and the top 10 core targets were *PIK3CA*, *PIK3R1*, *PIK3CB*, *SRC*, *JAK2*, *PTK2*, *PYPN11*, *MAPK3*, *STAT3*, and *AKT1* (Figures 2A,B). The PPI network of TCHMF1 had 164 nodes and 245 edges, with the top 10 core targets being: *ENPP1*, *CYP1A1*, *PTGS1*, *CYP1A2*, *AMPD3*, *CYP1B1*, *EPHX2*, *PTGS2*, *HPGDS* and *MAOB*. In TCHMF2, the PPI network had 168 nodes and 287 edges, with key targets such as *HK1*, *HK2*, *ENPP1*, *CYP1A1*, *AKR1B1*, and *HSD17B7*. The PPI network of TCHMF3 had 147 nodes and 398 edges, with core targets such as *CYP1A2*, *PTGS2,* and *COMT* (Figures 2C,D).

**Figure 3:**
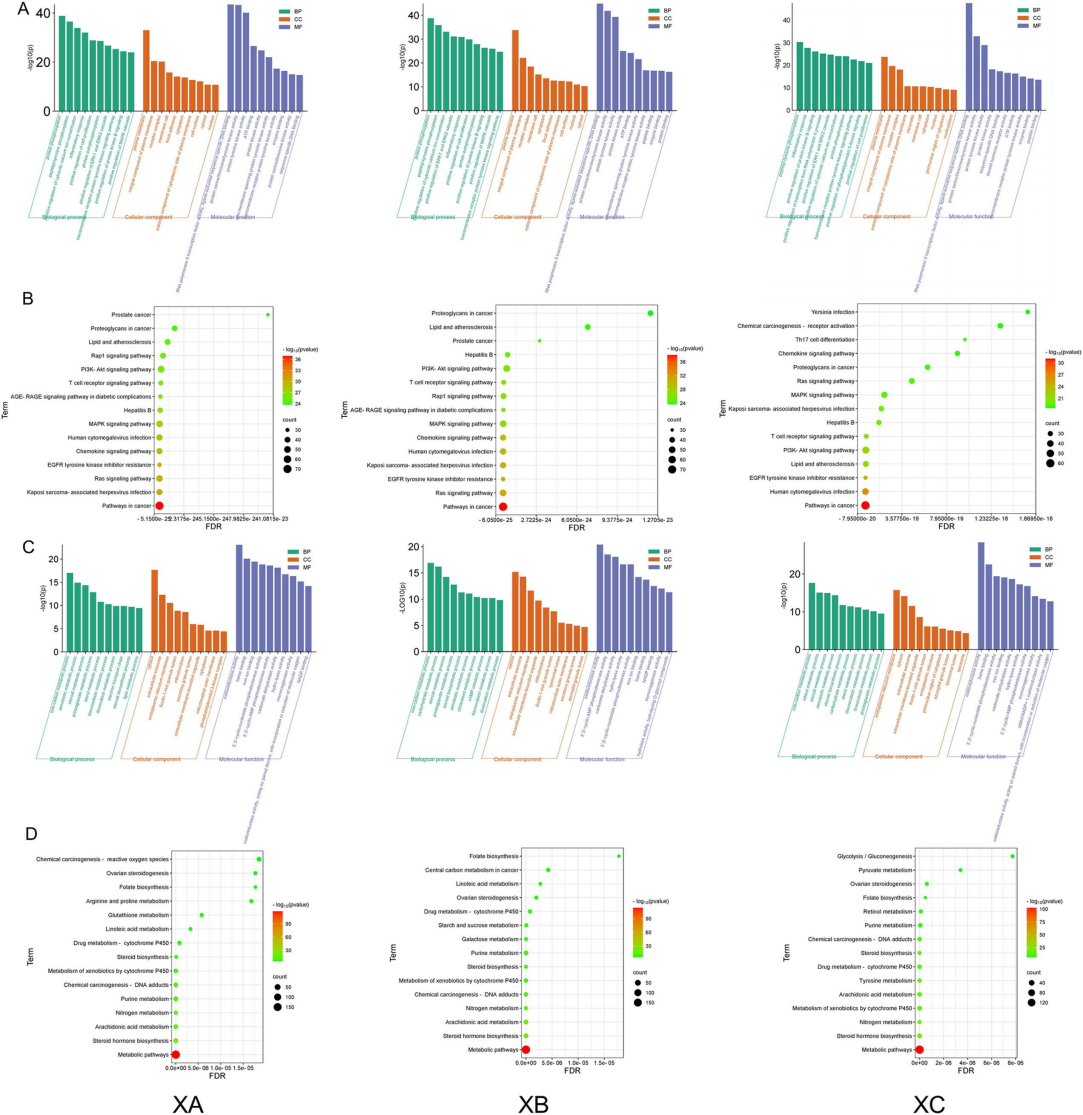
**(A)** GO enrichment analysis of active ingredients and immune common targets in Traditional Chinese herbal medicine formulas. **(B)** KEGG bubble chart showing active ingredient immune common target in Traditional Chinese herbal medicine formulas. **(C)** GO enrichment analysis of active ingredients and metabolism common targets in Traditional Chinese medicine formulas. **(D)** KEGG bubble chart showing active ingredients and metabolic common targets in Traditional Chinese medicine formulas.

The top 10 terms for each category of the GO enrichment analysis (biological process, cellular component, and molecular function for the active ingredient-immune/metabolic common targets of TCM compounds) were selected for visualization. The top 15 pathways identified by KEGG enrichment analysis for the active ingredient-disease common targets are displayed in Figure 3. Due to the high overlap between TCM compounds in TCHMC1 and TCHMC2, the GO enrichment analysis results for the active ingredient–immune common targets were similar between these two compounds. “Protein phosphorylation” and “plasma membrane” were the most significantly enriched terms in *BP*, *CC,* and *MF*, respectively, for compounds 1 and 2. However, for compound three, “peptidyl-tyrosine phosphorylation” was the most enriched term at the biological process level. Additionally, the number of functional genes in the *BP* and *CC* categories for compound three was lower than for compounds 1 and 2 (Figure 3A). In the KEGG enrichment analysis, pathways in cancer were the most abundant in immune-related pathways across all three TCHMCs. Kaposi sarcoma-associated herpesvirus infection and Ras signaling pathway were the second most enriched pathways for TCHMC1, TCHMC2, and TCHMC3 (Figure 3B). For metabolism-related targets, one-carbon metabolic process, cytosol, and oxidoreductase activity were the most significantly enriched GO terms across *BP*, *CC*, and *MF*. In compound three, however, “endoplasmic reticulum membrane” was the most abundant term at the cellular component level (Figure 3C). The most significantly enriched metabolic-related KEGG analysis across the three TCHMC metabolic pathways and steroid hormone biosynthesis followed by arachidonic acid metabolism in TCHMF1 and TCHMF2, and nitrogen metabolism in TCHMF3 (Figure 3D).

### The effect of adding traditional Chinese medicine compounds on the intestinal metabolites of weaned yaks

**Figure 4:**
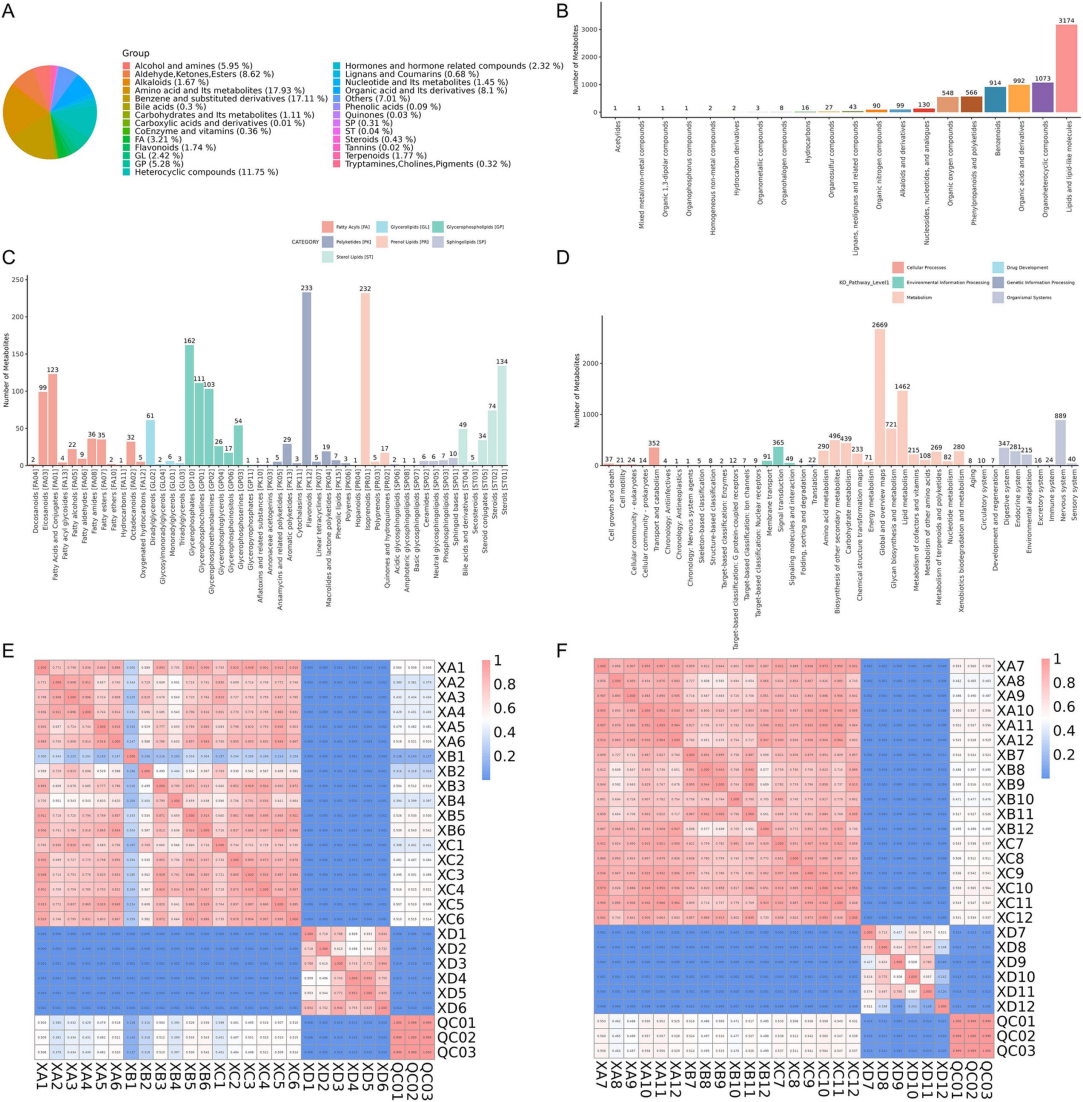
**(A)** Classification pie chart of metabolites in Class I. **(B)** Sample annotation of the Human Metabolome Database (HMDB) at the Superclass level. **(C)** Sample annotation of LIPID MAPS. **(D)** Sample annotation of the KEGG pathway database. **(E,F)** Sample correlation and quality control analysis.

The impact of incorporating TCHM compounds into the diet on the intestine metabolite composition of weaned yaks was analyzed by examining rectal contents from groups XA, XB, XC, and XD. A total of 10, 633, and 9,262 features were detected in the ESI+ (positive) and ESI (negative) ion modes, respectively. To explore the chemical classification of the identified metabolites, a pie chart was generated, reflecting the distribution and quantity of metabolites in each classification. In class I, amino acid and their metabolites constituted the largest portion (17.93%), followed by benzene and substituted derivatives (17.11%) and heterocyclic compounds (11.75%; Figure 4A).

The functions and classifications of identified metabolites were annotated using databases such as the HMDB, LIPID MAPS, and KEGG to further explore their functional characteristics. The HMDB annotations at the superclass level revealed that lipids and lipid-like molecules were the most abundant metabolite class (Figure 4B). The LIPID MAPS database, which contains biologically relevant lipid structures and annotations, identified glycerophospholipids as the most prevalent lipid class, with flavonoids [PK12] being the most abundant lipid subtype (Figure 4C). The KEGG pathway analysis revealed that pathways related to metabolism were most enriched, with global and overview maps(level 2)having the highest representation (Figure 4D). As the metabolome is highly sensitive to external influences and undergoes rapid changes, quality control (QC) is critical to ensuring stable and accurate results in metabolomic studies. Pearson correlation coefficients for the QC samples, based on the relative quantitative values of metabolites, were calculated as R^2^ > =0.999, indicating excellent stability throughout the detection process. The high-quality data met the requirements for further analysis. Additionally, correlation analysis revealed a strong correlation among the experimental groups, while the correlation between the experimental groups and control group was relatively low (Figures 4E,F).

**Figure 5:**
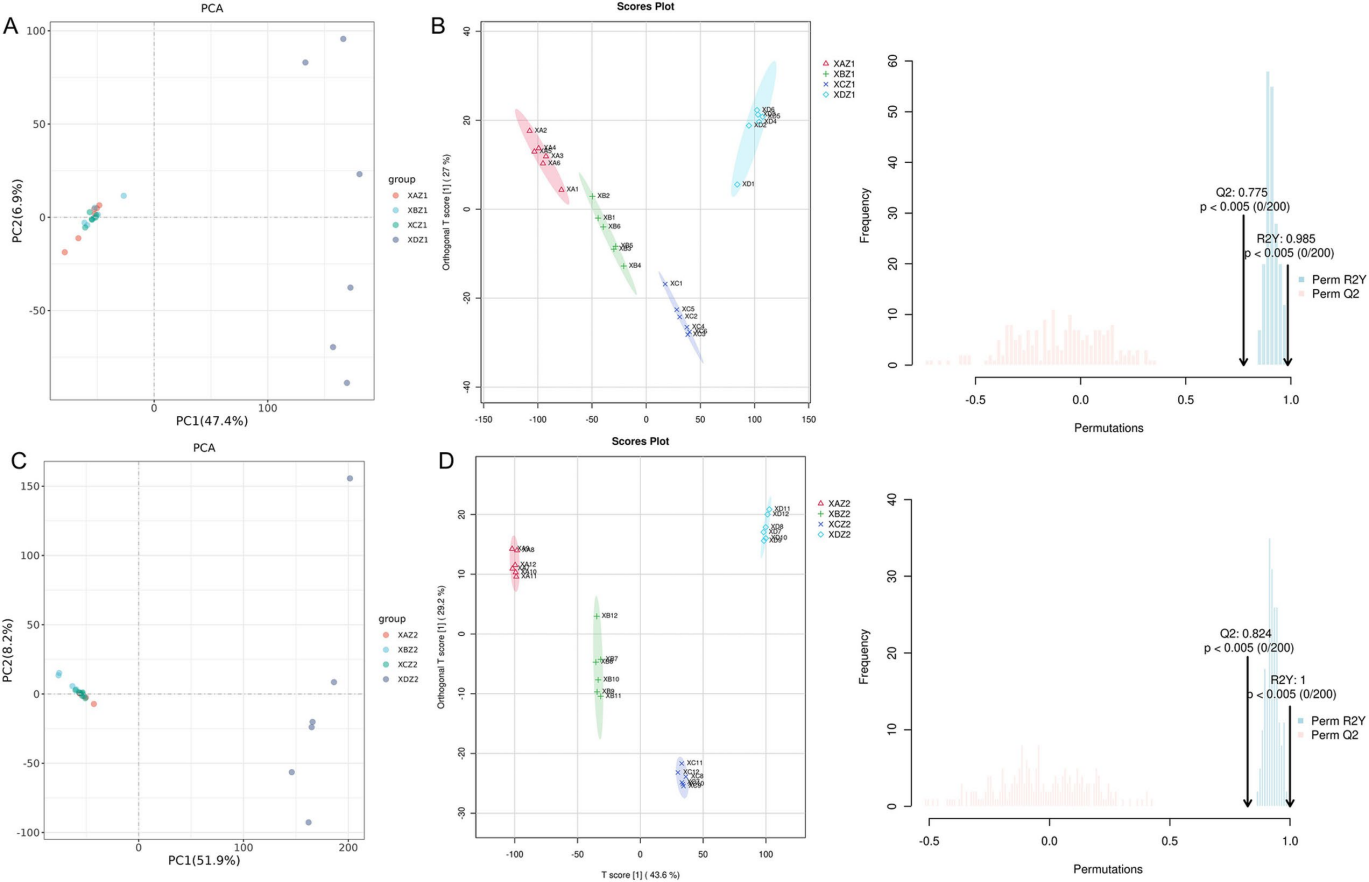
**(A)** Principal component analysis (PCA) of the total sample on the 15th day of the experiment. **(B)** Score plot of OPLS-DA of the total sample on the 15th day of the experiment. **(C)** PCA of the total sample on the 30th day of the experiment. **(D)** Score plot of OPLS-DA of the total sample on the 30th day of the experiment.

Principal component analysis (PCA) was conducted to classify the main new variables (principal components) based on their similarity, providing a global overview of the variations in metabolite data (28). The results revealed that data points from the groups supplemented with TCM formulas in the diet were clustered closely together, suggesting minimal metabolic differences between the experimental group samples. Furthermore, these variations tended to decrease over time. In contrast, data points from the control group were more dispersed and farther from the experimental groups (Figures 5A,C).

Figures 5B,D depict the score plots of orthogonal partial least squares discriminant analysis (OPLS-DA), which was performed to verify the differentiation of metabolites between the four groups. OPLS-DA maximized the differences among the groups and provided objective insights into group relationships, showing a clear separation and distinction between the control and the experimental groups. Additionally, as time progressed, the intragroup relationships became closer, whereas intergroup differences became more pronounced. The validation diagrams showed Q^2^ > 0.75 and *p* < 0.005, indicating that the models were robust and effectively identified differences between groups. The clustering heatmap analysis further highlighted a distinct separation in the gut metabolome between the control and experimental groups, supporting the results of the PCA and OPLS-DA.

### Adding traditional Chinese herbal medicine compounds to the diet caused significant changes in metabolites in weaned yaks

**Figure 6:**
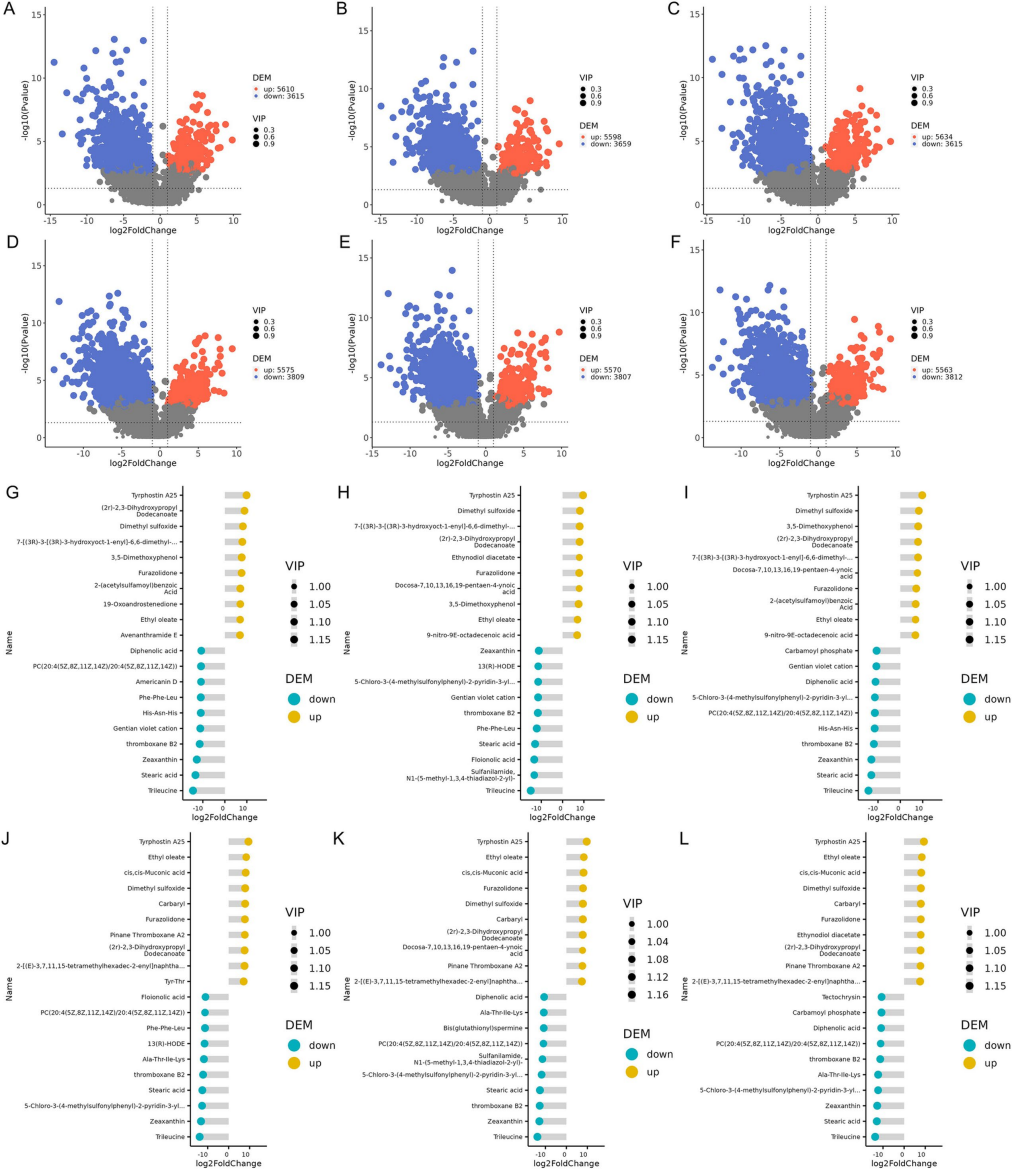
**(A–F)** Volcanic plot of differential metabolites. The horizontal axis represents the fold change of metabolites in different groups [log2 (fold change)] and the vertical axis represents the level of significance [− log10 (*p*-value)]. **(G–L)** Differential metabolite matchstick chart, blue representing downregulation and yellow representing upregulation; the length of the pole represents the size of log2 (fold change); the size of the dot represents the size of the VIP value.

Significant differential metabolites were screened using thresholds of VIP >1.0, FC > 2.0, or FC < 0.5 with *p*-value<0.05. In the samples collected on the 15th day of the experiment, 9,225 differential metabolites were identified in the XAZ1 group, 9,257 in the XBZI group, and 9,249 in the XCZ1 group compared to the XDZ1 group. At the end of the experiment cycle, 9,384 differential metabolites were detected in the XAZ2 group, 9,377 in the XBZ2 group, and 9,375 in the XCZ2 group, compared to the XDZ2 group. The specific number of upregulated and downregulated metabolites is presented in Supplementary Table 16. Volcano plots provided an intuitive representation of the overall distribution of differential metabolites (Figures 6A–F).

Matchstick diagrams were generated based on the differential metabolites identified in each group comparison, the top 20 upregulated and downregulated metabolites. On the 15th day, *Tyrphostin A25*, *Dimethyl sulfoxide,* and *(2r)-2,3-Dihydroxypropyl Dodecanoate* were significantly upregulated in all experimental groups, whereas *Trileucine*, *Stearic acid,* and *thromboxane B2* were significantly downregulated. *19-Oxoandrostenedione*, and *Avenanthramide E* markedly increased in the XAZ1 group, while *Americanin D* decreased (Figure 6G). *13(R)-HODE and Floionolic acid* significantly decreased in the group XBZ1 (Figure 6H), and Carbamoyl *phosphate was* decreased in the XCZ1 group (Figure 6I). In the samples collected on the 30th day, *Tyrphostin A25*, *Ethyl oleate,* and *Zeaxanthin* were significantly upregulated across all experimental groups, whereas *Trileucine*, *cis,cis-Muconic,* and *Stearic acid* were significantly downregulated. *Tyr-Thr* showed a notable increase, whereas *13(R)-HODE*, *Phe-Phe-Leu,* and *Floionolic acid* were decreased in the XAZ2 group (Figure 6J); *Docosa-7,10,13,16,19-pentaen-4-ynoic acid* was significantly increased, whereas *Bis(glutathionyl)spermine* decreased in the XBZ2 group (Figure 6K), In the XCZ2 group, *Ethynodiol diacetate* was significantly enriched, whereas *Tectochrysin* and *Carbamoyl phosphate* were decreased (Figure 6L).

**Figure 7:**
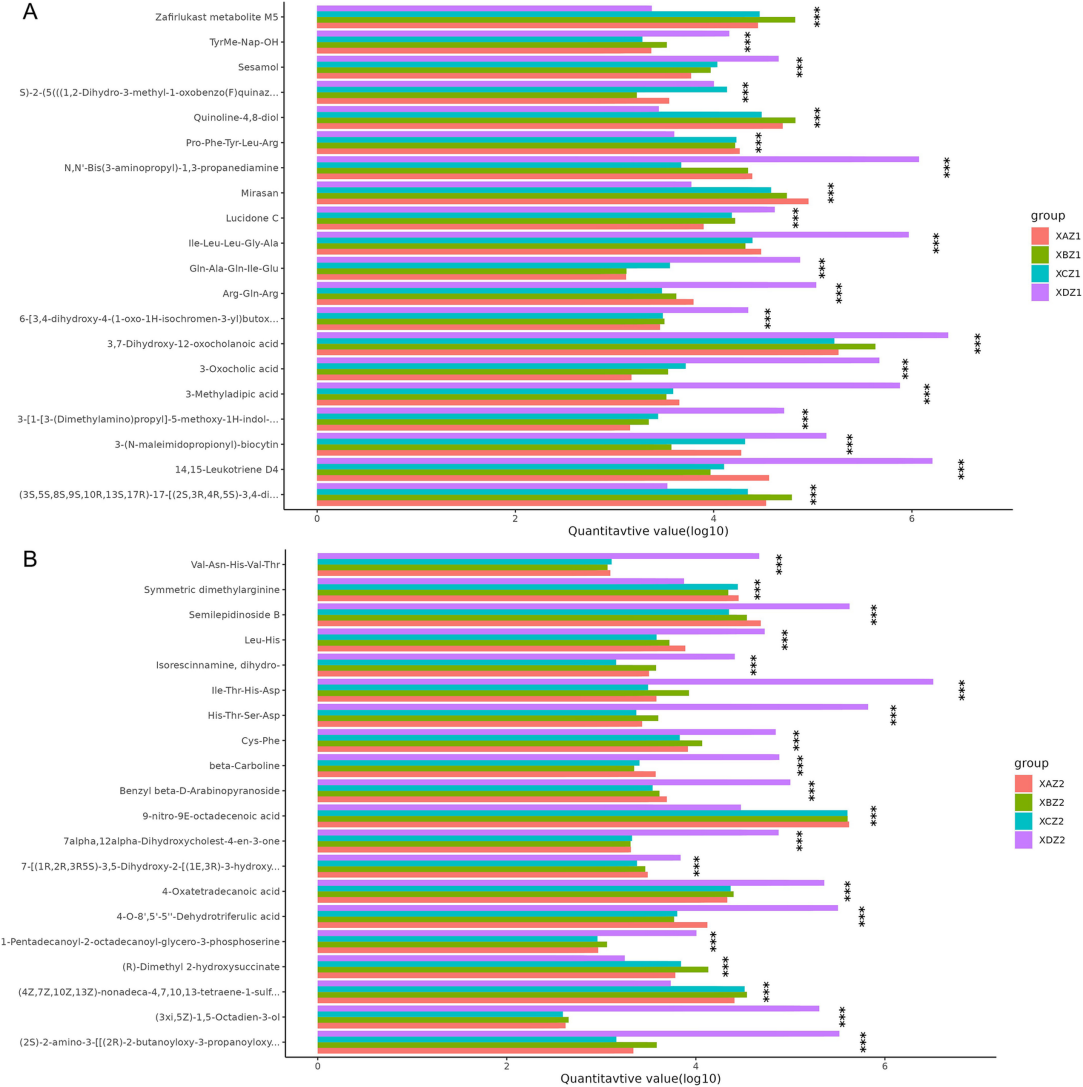
ANOVA of significant differences across the four groups. **(A)** Samples collected on the 15th day of the experiment. **(B)** Samples collected on the 30th day of the experiment.

ANOVA was conducted to identify metabolites with significant differences among the four groups. The top 20 differential metabolites with the most significant differences are shown in Figure 7. In the XDZ1 group compared to the three experimental groups, metabolites such as *4,15*-*Leukotriene D4*, *3*-*Methyladipic acid*, and *3*-*Oxocholic acid* were significantly enriched, while *Mirasan*, *Zafirlukast metabolite M5,* and *Quinoline−4,8*-*diol* were reduced. In the XAZ1 group, (*2R*)*-O-phospho-3-sulfolactic acid* and *Agathisflavone were* decreased whereas *N,N′-Bis(3-aminopropyl)-1,3-propanediamine* and *Cl-amidine* were significantly decreased in the XCZ1 group. Conversely, *N-(octadecanoyl)-sphing-4-enine-1-phosphocholine* and *2-Oxopropyl-CoM* were increased (Figure 7A). In the XDZ2 group, *(3xi,5Z)-1,5-Octadien*-*3*-*ol*, *4*-*O*-*8′,5′-5′′*-*Dehydrotriferulic acid* and *4*-*Oxatetradecanoic acid* were more abundant, while *(R)-Dimethyl 2*-*hydroxysuccinate*, *9*-*nitro*-*9E*-*octadecenoic acid,* and *Symmetric dimethylarginine* were less abundant. *Cys-Phe* was found in the XBZ2 group, and *Leu-Met-OH* was reduced in the XCZ2 group (Figure 7B).

### The impact of adding traditional Chinese herbal medicine compounds to the diet on the KEGG metabolic pathways

**Figure 8:**
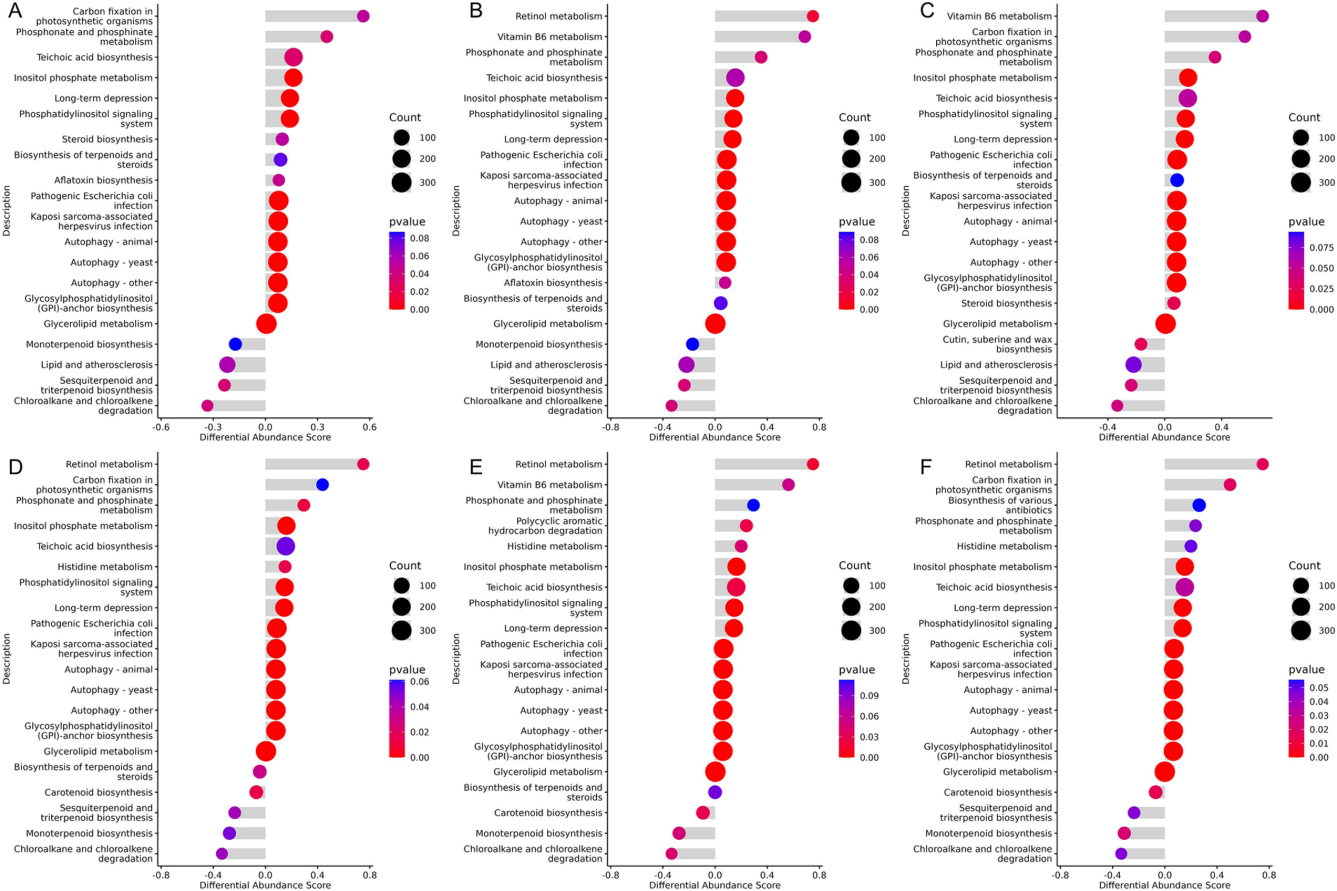
KEGG differential abundance score (DA Score) plot. The horizontal axis represents the differential abundance score, and the vertical axis represents the differential pathway names. **(A)** Group XAZ1 VS. XDZ1. **(B)** Group XBZ1 VS. XDZ1. **(C)** Group XCZ1 VS. XDZ1. **(D)** Group XAZ2 VS. XDZ2. **(E)** Group XBZ2 VS. XDZ2. **(F)** Group XCZ2 VS. XDZ2.

The Kyoto Encyclopedia of Genes and Genomes (KEGG) is a public database used for systematically analyzing metabolic pathways and the functions of gene products within cells. In this study, the KEGG differential abundance DA score was utilized to assess the overall changes in metabolites within specific metabolic pathways. A DA score greater than 0 indicates an upregulation of the expression trend for all annotated metabolites in the pathway, while a score less than 0 indicates a downregulation. The results revealed that pathways such as Teichoic acid biosynthesis, *Phosphonate and phosphonate metabolism*, long-term depression, Inositol phosphate metabolism, and several autophagy-related pathways were significantly upregulated in groups supplemented with Chinese herbal compounds compared to the control group. Carbon fixation in photosynthetic organisms showed an increase in the XAZ1 and XCZ1 groups, and vitamin B6 metabolism was upregulated in the XBZ1 and XCZ1 groups. In contrast, chloroalkane and chloroalkene degradation, sesquiterpenoid and triterpenoid biosynthesis, and lipid and atherosclerosis pathways were downregulated in the groups with dietary supplementation, as shown in Figures 8A–C. In the later stage of the experiment, retinol metabolism was notably upregulated in the groups receiving dietary supplementation along with pathways such as phosphonate and phosphonate metabolism, histidine metabolism, inositol phosphate metabolism, phosphatidylinositol signaling system, glycosylphosphatidylinositol (GPI)-anchor biosynthesis, and several pathways associated with autophagy. Additionally, vitamin B6 metabolism was significantly enriched in the XBZ2 group. Conversely, chloroalkane and chloroalkene degradation, monoterpenoid biosynthesis, and carotenoid biosynthesis were significantly downregulated in the experimental groups compared to the control group. Sesquiterpenoid and triterpenoid biosynthesis also decreased in the XAZ2 and XCZ2 groups (Figures 8D–F).

## Discussion

Network pharmacology is a systematic research method that integrates laboratory findings, clinical inquiries, and data processing to support drug discovery and development (3). It is particularly effective for studying the complex interactions between TCM and various diseases (29). In this study, the primary component genes of the herbal formula were analyzed for their interaction with immune and metabolic genes to explore their therapeutic efficacy. The protein–protein interaction (PPI) network for immune-related genes in TCHMF1 contained 179 nodes and 540 edges, TCHMF2 had 189 nodes and 567 edges, and TCHMF3 had 157 nodes and 435 edges. Similarly, the PPI networks for I metabolic-related genes included 164 nodes and 245 edges in TCHMF1, 168 nodes and 287 edges in TCHMF2, and 147 nodes and 398 edges in TCHMF3. The core target genes from these networks may be involved in the therapeutic effects of the three TCHMFs.

The core genes in the PPI network between TCM and immune-related genes are primarily involved in regulating cell proliferation and division. For instance, *PIK3R1* is known for its tumor-suppressing potential, which is associated with various cancers due to its mutations. The *PIK3CA* gene encodes a subunit of the *PI3K* enzyme, which is linked to cell survival by activating anti-apoptotic signals (30). Another significant gene, *Src*, encodes a protein tyrosine kinase crucial for cell growth, division migration, and survival. *Src* is also implicated in malignant transformation and tumorigenesis (31). The protein encoded by *PTPN11* regulates several cellular processes, including growth, differentiation, and the mitotic cycle. Mutations in *PTPN11* are linked to Noonan syndrome and acute myeloid leukemia. Additionally, *STAT3*, a transcription factor, regulates genes associated with cancer cell survival, proliferation, angiogenesis, invasion, metastasis, drug resistance, and immune evasion. Dysregulation of *STAT3*, whether over-activated or inactivated, is linked to diseases, underscoring the importance of its precise regulation (32). Therefore, the Chinese herbs in the TCHMFs may exert their effects by modulating cellular life processes, immune functions, and inflammation-related pathways.

The core genes involved in the metabolic-related gene protein interaction network are associated with pathways regulating various metabolic diseases. ENPP1 is expressed in many tissues and is vital for purinergic signaling, which regulates cardiovascular, neurological, immune, musculoskeletal, hormonal, and hematological functions in mammals. A study by Chen et al. (51) demonstrated that in a mouse model, the red blood cell *ENT1-AMPD3* axis is a key energy regulator in the intracellular purinergic hypoxia compensation response, enhancing the extracellular adenosine-mediated rapid energy supply. This mechanism is particularly critical for yaks, especially calves with incomplete physical development, living in high-altitude environments. *CYP1A2*, a member of the *CYP1* family, is predominantly expressed in the liver and accounts for approximately 10 to 15% of the total liver CYP content. It plays a major role in the metabolism of 10 to 15% of drugs. *CYP1B1* is involved in regulating metabolic pathways, including steroid hormone, fatty acid, vitamin, and melatonin metabolism, which are linked to the development of metabolic diseases such as metabolic syndrome, insulin resistance, hepatic steatosis, inflammation, and endothelial dysfunction. Studies suggest that sEH also contributes to obesity-induced intestinal barrier dysfunction and bacterial translocation, a key factor in the pathogenesis of obesity-related diseases (33). The *PTGS2* gene, encoding cyclooxygenase-2 (COX2), plays a crucial role in immune cell infiltration and enriched signaling pathways (34). A study by Meriwether et al. (53) highlighted that macrophage (COX2) deficiency exacerbates intestinal inflammation similar to inflammatory bowel disease (IBD). Macrophage COX2 enhances exocytosis and dependent reprogramming, thereby promoting intestinal epithelial repair. *AKR1B1* is involved in a complex network of signaling pathways, including *miR-21*-mediated inflammatory response cell cycle regulation, epithelial-to-mesenchymal transition, cell survival, and apoptosis mechanisms. *AKR1B10 a* (NADPH)-dependent reductase is highly expressed in the epithelial cells of the stomach and intestine but downregulated in gastrointestinal cancer and inflammatory bowel disease (35). These metabolic pathways are intricately linked to the growth, development, immune function, and gut health of calves, emphasizing their importance in promoting healthy physiological development.

GO enrichment analysis of the active ingredient immune common target of the compound revealed that “protein phosphorylation” and “plasma membrane” were the most significantly enriched categories at three levels of the GO. “Protein phosphorylation” is a crucial regulatory factor for protein and cellular functions (36). As the most abundant post-translational modification of proteins, phosphorylation regulates numerous cellular processes, including cell growth, differentiation, apoptosis, and cell signaling under normal conditions. However, disruptions in the phosphorylation pathway can lead to severe consequences, particularly in the development of diseases, such as cancer (37). The “plasma membrane” plays a crucial role in providing a biophysical and biochemical platform for immune cells, facilitating the initiation of the signaling cascades and immune responses that defend against foreign pathogens or tumor cells (38). The immune-relative KEGG pathways identified in this analysis were predominantly associated with tumors, including the Ras signaling pathway and the Kaposi sarcoma-associated herpesvirus infections, as well as pathways linked to metabolic and cardiovascular diseases.

The inclusion of TCM compounds in the diet significantly affected the composition of intestinal metabolites in weaned yaks. Cellular signal transduction and cellular metabolism are intricately connected, signaling pathways can regulate cellular metabolism, and recent studies indicate that metabolism, in turn, can influence signaling. Tyrphostin A25, a member of the tyrosine kinase inhibitor family, binds competitively and inhibits the GTPase activity of epidermal growth factor receptors and transduction proteins, inducing apoptosis in human leukemia cell lines. G. Partik et al. investigated its effects on colorectal tumor cells and found that it inhibits DNA synthesis and induces apoptosis, demonstrating therapeutic potential (39). At low concentrations, DMSO exhibits various properties, including anti-inflammatory, analgesic, diuretic, vasodilatory, antiplatelet aggregation, radiation protection, and muscle relaxation effects. Additionally, DMSO is a potent scavenger of hydroxyl radicals, enhancing its antioxidant effects in ischemic and inflammatory conditions (40–42). Clinically, DMSO has been shown to regulate the immune regulation response, reduce inflammation, and prevent amyloid fibrillar degeneration (43). Furthermore, DMSO is an effective antioxidant and anti-inflammatory agent, which contributes to reducing oxidative stress markers. Reduced thromboxane A2 (TXA2) levels observed in this study suggest a diminished thrombogenic potential, correlating with improved intestinal and systemic health (44, 45). The increase in these metabolites suggests that TCM can enhance the physical fitness of calves through its anti-inflammatory and immune-regulating properties. In the first stage of the experiment, metabolites related to the biosynthesis of unsaturated fatty acids, such as stearic acid, were significantly reduced. An increase in thromboxane B2 (TXB2) may lead to thrombosis and increase the risk of atherothrombotic events (44, 46). However, the experimental group showed a reduction in TXB2 levels, which lowered the risk of such events (45). For humans, zeaxanthin known for its photoprotective, anti-inflammatory, and brain-boosting properties has potent antioxidant effects that provide health effects throughout the lifecycle (47). Gehan El Akabawy et al. demonstrated that zeaxanthin exerts a protective effect against acetic acid-induced colitis in rats by modulating pro-inflammatory cytokines and oxidative stress (48). The increase in zeaxanthin in the intestinal metabolites of the experimental groups suggests that Chinese herbal medicine may offer protective effects on the intestines. Vitamin B6 metabolism was elevated in the XBZ1 and XCZ1 groups with its active form, pyridoxal 5′-phosphate (PLP), acting as a cofactor in over 150 enzymatic reactions. One possible mechanism is that vitamin B6 may be mobilized to the inflammation site, where it participates in metabolic pathways with immunomodulatory effects. Supplementation of vitamin B6 has been shown to improve immune functions in patients with vitamin B6 deficiency and in the experimental animals (49).

The addition of TCM formulas to the diet leads to significant downregulation of several KEGG metabolic pathways, such as those involved in chloroalkane and chloroalkane degradation, sesquiterpenoid and triterpenoid biosynthesis, and lipid and atherosclerosis. Liu et al. reported that the metabolic pathways related to chloroalkane and chloroalkane degradation were significantly upregulated in the gut microbiota of patients with chronic kidney disease. Fungi, particularly from the phyla *Ascomycota* and *Basidiomycota*, are known to produce structurally diverse terpenoids, including sesquiterpenes, diterpenes, and triterpenoids. The observed downregulation of these metabolic pathways may reflect a decrease in the abundance of fungi involved in terpenoid production (50). Additionally, the reduction in lipid and atherosclerotic-related pathways suggests that TCM may play a role in regulating fat metabolism and reducing the risk of lipid-related metabolic diseases.

On the other hand, the experimental groups showed upregulation in vitamin A metabolism and autophagy-related pathways. Vitamin A is crucial for several physiological processes, including embryogenesis, vision, cell proliferation and differentiation, immune regulation, and glucose and lipid metabolism. Enhanced vitamin A metabolism, observed in experimental groups, implies improved epithelial repair and immune resilience. Supplementation with vitamin A or its precursors may provide targeted support during weaning stress to promote gut and systemic health. Moreover, autophagy, a cellular catabolic process, plays a critical role in maintaining the intestinal epithelium. Many autophagy-related genes have been linked to intestinal diseases. In addition, autophagy itself is essential for intestinal homeostasis that repairs and supports intestinal barrier function by managing cellular stress and also influences intestinal stem cells, impacting their metabolism, proliferation, and regeneration capacity. In summary, Chinese herbal medicine can enhance immunity and improve intestinal function in calves by influencing gut microbiota, metabolic pathways, fat metabolism, and other related processes.

## Conclusion

This study combines network pharmacology analysis with yak gut metabolomics to explore the effects of three TCM formulas on weaned yaks. Network pharmacology analysis revealed that these TCM formulas interact with multiple tumor-related immune genes, which are closely linked to cell growth processes. The interaction between metabolic-related genes and proteins identified top targets involved in purine metabolism, blood cell metabolism, inflammatory responses, and other pathways critical for growth, immune regulation, and antioxidant function in calves. Weaning is a crucial period in the life of yaks involving a physiological change. During this time, the intake of TCM significantly alters the abundance of various intestinal metabolites, such as DMSO, tyrphostin A25, and zeaxanthin. Several metabolic pathways, including those related to autophagy, lipid and atherosclerosis, and vitamin A metabolism, were upregulated in the experimental groups. These findings suggest that TCM may improve the physical fitness of weaned yaks and reduce the risk of diarrhea by regulating cell growth, improving immune function, and enhancing anti-inflammatory and antioxidant responses. This study provides preliminary insights into the pharmacological mechanism of TCM in preventing diarrhea and improving physical fitness in weaned yaks, which is valuable for further evaluation of the efficacy of these formulas. Additionally, the major limitations of this study include a small sample size and short experimental duration. Future research should explore larger cohorts and extend the study period to confirm the long-term benefits of TCM supplementation. Future studies will explore the long-term effects of TCM supplementation on yak productivity and health, with a particular focus on gut microbiota dynamics and host–microbe interactions.

## Data Availability

The original contributions presented in the study are included in the article/Supplementary material, further inquiries can be directed to the corresponding authors.

## Footnotes

^1^ https://bio-cloud.aptbiotech.com/

^2^ http://www.bioinformatics.com.cn/

^3^ https://hmdb.ca/metabolites

^4^ http://www.lipidmaps.org/

## Supplementary material

SUPPLEMENTARY FIGURE S1 TMC-ingredient-target interaction network maps. **(A)** Traditional Chinese Medicine formula I, **(B)** Traditional Chinese Medicine formula II, **(C)** Traditional Chinese Medicine formula III.
