# Supplementary material for: Joint exploration of network pharmacology and metabolomics on the effects of traditional Chinese medicine compounds in weaned yaks
Source: Front Vet Sci. 2025 Jan 13;11:1511311. doi: 10.3389/fvets.2024.1511311 (PMC11770994; doi:10.3389/fvets.2024.1511311)
Supplement: SUPPLEMENTARY FIGURE S1 — TMC-ingredient-target interaction network maps. (A) Traditional Chinese Medicine formula I, (B) Traditional Chinese Medicine formula II, (C) Traditional Chinese Medicine formula III. [file Presentation_1.pptx]

## Slide 1
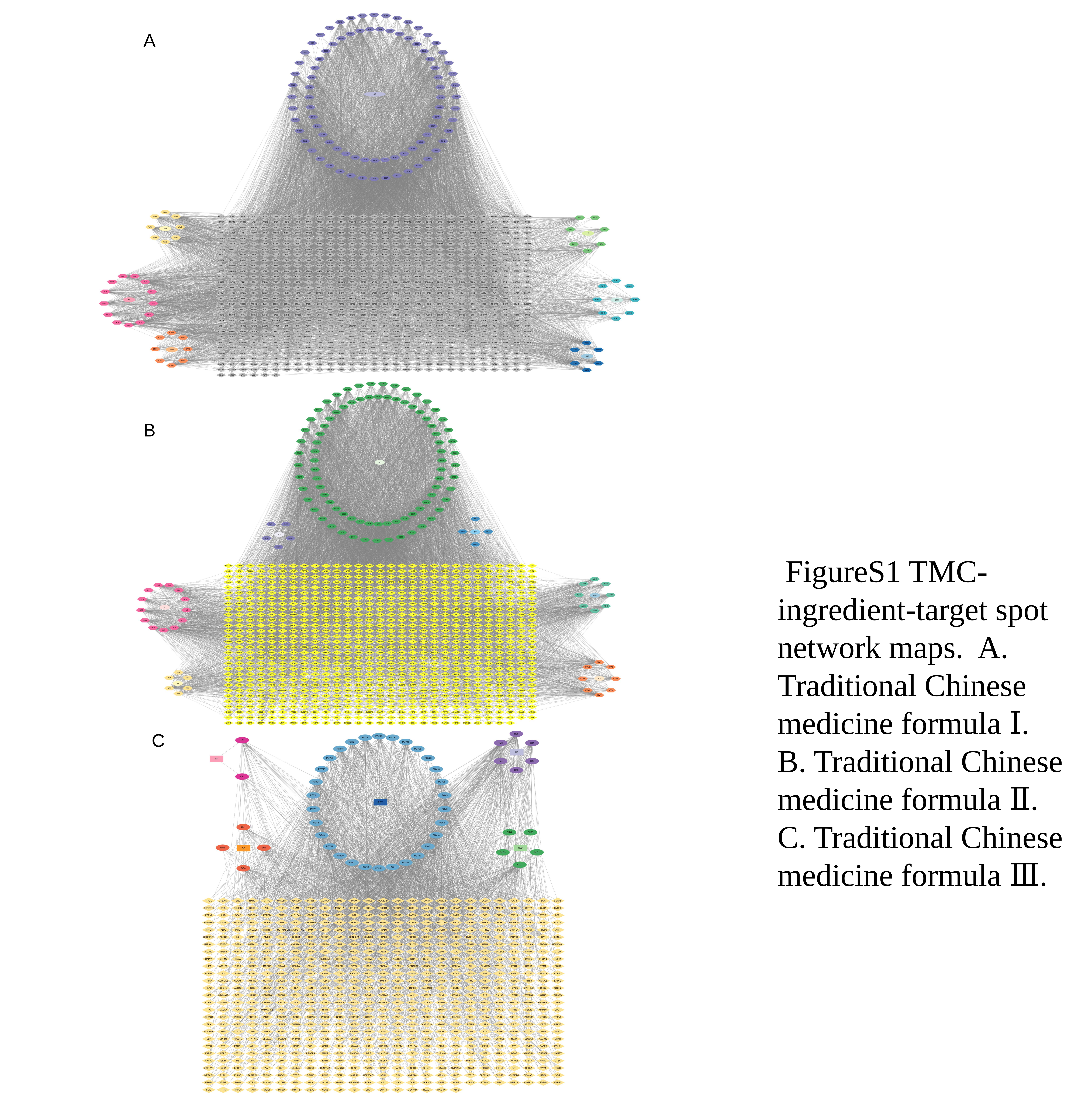

A
B
 FigureS1 TMC-ingredient-target spot network maps. A. Traditional Chinese medicine formula Ⅰ. B. Traditional Chinese medicine formula Ⅱ. C. Traditional Chinese medicine formula Ⅲ.
C
