# Supplementary material for: Joint exploration of network pharmacology and metabolomics on the effects of traditional Chinese medicine compounds in weaned yaks
Source: Front Vet Sci. 2025 Jan 13;11:1511311. doi: 10.3389/fvets.2024.1511311 (PMC11770994; doi:10.3389/fvets.2024.1511311)
Supplement: Supplementary file 2 [file Table_1.docx]

| **Time** | **A%** | **B%** |
| --- | --- | --- |
| 0 | 98 | 2 |
| 1.5 | 98 | 2 |
| 3 | 0 | 100 |
| 10 | 0 | 100 |
| 10.1 | 98 | 2 |
| 11 | 98 | 2 |
| 12 | 98 | 2 |

Table1 Chromatographic gradient elution program

| **Traditional Chinese Medicine** | **Identifier** | **Full name** | **OB(%)** | **DL** |
| --- | --- | --- | --- | --- |
| Coptis chinensis | HL1 | berberine | 36.86 | 0.78 |
| Coptis chinensis | HL2 | Obacunone | 43.29 | 0.77 |
| Coptis chinensis | HL3 | berberrubine | 35.74 | 0.73 |
| Coptis chinensis | HL4 | epiberberine | 43.09 | 0.78 |
| Coptis chinensis | HL5 | (R)-Canadine | 55.37 | 0.77 |
| Coptis chinensis | HL6 | Berlambine | 36.68 | 0.82 |
| Coptis chinensis | HL7 | Magnograndiolide | 63.71 | 0.19 |
| Coptis chinensis | HL8 | Palmidin A | 35.36 | 0.65 |
| Coptis chinensis | HL9 | palmatine | 64.6 | 0.65 |
| Coptis chinensis | HL10 | quercetin | 46.43 | 0.28 |
| Coptis chinensis | HL11 | coptisine | 30.67 | 0.86 |
| Coptis chinensis | HL12 | Worenine | 45.83 | 0.87 |
| Coptis chinensis | HL13 | Moupinamide | 86.71 | 0.26 |

Table 2 Basic information on the ingredients of Coptis chinensis (HL)

| **Traditional Chinese Medicine** | **Identifier** | **Full name** | **OB(%)** | **DL** |
| --- | --- | --- | --- | --- |
| Chinese pulsatilla | BTW1 | ZINC01615307 | 56.38 | 0.87 |
| Chinese pulsatilla | BTW2 | Sitosteryl acetate | 40.39 | 0.85 |
| Chinese pulsatilla | BTW3 | Mairin | 55.38 | 0.78 |
| Chinese pulsatilla | BTW4 | Stigmasterol | 43.83 | 0.76 |
| Chinese pulsatilla | BTW5 | beta-sitosterol | 36.91 | 0.75 |
| Chinese pulsatilla | BTW6 | LAN | 42.12 | 0.75 |
| Chinese pulsatilla | BTW7 | isorhamnetin | 49.6 | 0.31 |
| Chinese pulsatilla | BTW8 | Aureusidin | 53.42 | 0.24 |

Table 3 Basic information on the ingredients of Chinese pulsatilla (BTW)

| **Traditional Chinese Medicine** | **Identifier** | **Full name** | **OB(%)** | **DL** |
| --- | --- | --- | --- | --- |
| dark plum | WM1 | (2R)-5,7-dihydroxy-2-(4-hydroxyphenyl)chroman-4-one | 42.36 | 0.21 |
| dark plum | WM2 | beta-sitosterol | 36.91 | 0.75 |
| dark plum | WM3 | kaempferol | 41.88 | 0.24 |
| dark plum | WM4 | Stigmasterol | 43.83 | 0.76 |
| dark plum | WM5 | campest-5-en-3beta-ol | 37.58 | 0.71 |
| dark plum | WM6 | Methyl arachidonate | 46.9 | 0.23 |
| dark plum | WM7 | CLR | 37.87 | 0.68 |
| dark plum | WM8 | quercetin | 46.43 | 0.28 |

Table 4 Basic information on the ingredients of dark plum (WM)

| **Traditional Chinese Medicine** | **Identifier** | **Full name** | **OB(%)** | **DL** |
| --- | --- | --- | --- | --- |
| myrobalan | HZ1 | ellagic acid | 43.06 | 0.43 |
| myrobalan | HZ2 | chebulic acid | 72 | 0.32 |
| myrobalan | HZ3 | ellipticine | 30.82 | 0.28 |
| myrobalan | HZ4 | Peraksine | 82.58 | 0.78 |
| myrobalan | HZ5 | (R)-(6-methoxy-4-quinolyl)-[(2R,4R,5S)-5-vinylquinuclidin-2-yl]methanol | 55.88 | 0.4 |
| myrobalan | HZ6 | Cheilanthifoline | 46.51 | 0.72 |

Table 5 Basic information on the ingredients of myrobalan (HZ)

| **Traditional Chinese Medicine** | **Identifier** | **Full name** | **OB(%)** | **DL** |
| --- | --- | --- | --- | --- |
| Rheum tibeticum | ZDH1 | EUPATIN | 50.8 | 0.41 |
| Rheum tibeticum | ZDH2 | Mutatochrome | 48.64 | 0.61 |
| Rheum tibeticum | ZDH3 | Physciondiglucoside | 41.65 | 0.63 |
| Rheum tibeticum | ZDH4 | rhein | 47.07 | 0.28 |
| Rheum tibeticum | ZDH5 | Toralactone | 46.46 | 0.24 |
| Rheum tibeticum | ZDH6 | Emodin-1-O-beta-D-glucopyranoside | 44.81 | 0.8 |
| Rheum tibeticum | ZDH7 | palmidin A | 32.45 | 0.65 |
| Rheum tibeticum | ZDH8 | beta-sitosterol | 36.91 | 0.75 |

Table 6 Basic information on the ingredients of Rheum tibeticum (ZDH)

| **Traditional Chinese Medicine** | **Identifier** | **Full name** | **OB(%)** | **DL** |
| --- | --- | --- | --- | --- |
| plantain seed | CQZ1 | (4aS,6aR,6aS,6bR,8aR,10R,12aR,14bS)-10-hydroxy-2,2,6a,6b,9,9,12a-heptamethyl-1,3,4,5,6,6a,7,8,8a,10,11,12,13,14b-tetradecahydropicene-4a-carboxylic acid | 32.03 | 0.76 |
| plantain seed | CQZ2 | Dinatin | 30.97 | 0.27 |
| plantain seed | CQZ3 | sitosterol | 36.91 | 0.75 |
| plantain seed | CQZ4 | Dihydrotricetin | 58.12 | 0.28 |
| plantain seed | CQZ5 | Hypolaetin | 33.24 | 0.28 |
| plantain seed | CQZ6 | quercetin | 46.43 | 0.28 |

Table 7 Basic information on the ingredients of plantain seed (CQZ)

| **Traditional Chinese Medicine** | **Identifier** | **Full name** | **OB(%)** | **DL** |
| --- | --- | --- | --- | --- |
| Radix liquiritiate | GC1 | 5,7-dihydroxy-3-(4-methoxyphenyl)-8-(3-methylbut-2-enyl)chromone | 30.49 | 0.41 |
| Radix liquiritiate | GC2 | icos-5-enoic acid | 30.7 | 0.2 |
| Radix liquiritiate | GC3 | gadelaidic acid | 30.7 | 0.2 |
| Radix liquiritiate | GC4 | (2S)-2-[4-hydroxy-3-(3-methylbut-2-enyl)phenyl]-8,8-dimethyl-2,3-dihydropyrano[2,3-f]chromen-4-one | 31.79 | 0.72 |
| Radix liquiritiate | GC5 | Isotrifoliol | 31.94 | 0.42 |
| Radix liquiritiate | GC6 | Phaseolinisoflavan | 32.01 | 0.45 |
| Radix liquiritiate | GC7 | Kanzonol F | 32.47 | 0.89 |
| Radix liquiritiate | GC8 | DFV | 32.76 | 0.18 |
| Radix liquiritiate | GC9 | licorice glycoside E | 32.89 | 0.27 |
| Radix liquiritiate | GC10 | Licocoumarone | 33.21 | 0.36 |
| Radix liquiritiate | GC11 | 3,22-Dihydroxy-11-oxo-delta(12)-oleanene-27-alpha-methoxycarbonyl-29-oic acid | 34.32 | 0.55 |
| Radix liquiritiate | GC12 | Sigmoidin-B | 34.88 | 0.41 |
| Radix liquiritiate | GC13 | 2-[(3R)-8,8-dimethyl-3,4-dihydro-2H-pyrano[6,5-f]chromen-3-yl]-5-methoxyphenol | 36.21 | 0.52 |
| Radix liquiritiate | GC14 | (2S)-7-hydroxy-2-(4-hydroxyphenyl)-8-(3-methylbut-2-enyl)chroman-4-one | 36.57 | 0.32 |
| Radix liquiritiate | GC15 | sitosterol | 36.91 | 0.75 |
| Radix liquiritiate | GC16 | glycyroside | 37.25 | 0.79 |
| Radix liquiritiate | GC17 | HMO | 38.37 | 0.21 |
| Radix liquiritiate | GC18 | 7-Acetoxy-2-methylisoflavone | 38.92 | 0.26 |
| Radix liquiritiate | GC19 | Licoisoflavone B | 38.93 | 0.55 |
| Radix liquiritiate | GC20 | (E)-1-(2,4-dihydroxyphenyl)-3-(2,2-dimethylchromen-6-yl)prop-2-en-1-one | 39.62 | 0.35 |
| Radix liquiritiate | GC21 | Inflacoumarin A | 39.71 | 0.33 |
| Radix liquiritiate | GC22 | licochalcone a | 40.79 | 0.29 |
| Radix liquiritiate | GC23 | (-)-Medicocarpin | 40.99 | 0.95 |
| Radix liquiritiate | GC24 | Glycyrrhiza flavonol A | 41.28 | 0.6 |
| Radix liquiritiate | GC25 | Licoisoflavone | 41.61 | 0.42 |
| Radix liquiritiate | GC26 | kaempferol | 41.88 | 0.24 |
| Radix liquiritiate | GC27 | 7-Methoxy-2-methyl isoflavone | 42.56 | 0.2 |
| Radix liquiritiate | GC28 | Eurycarpin A | 43.28 | 0.37 |
| Radix liquiritiate | GC29 | 3'-Hydroxy-4'-O-Methylglabridin | 43.71 | 0.57 |
| Radix liquiritiate | GC30 | 2-(3,4-dihydroxyphenyl)-5,7-dihydroxy-6-(3-methylbut-2-enyl)chromone | 44.15 | 0.41 |
| Radix liquiritiate | GC31 | Isoglycyrol | 44.7 | 0.84 |
| Radix liquiritiate | GC32 | Glepidotin A | 44.72 | 0.35 |
| Radix liquiritiate | GC33 | Isolicoflavonol | 45.17 | 0.42 |
| Radix liquiritiate | GC34 | Glyasperin C | 45.56 | 0.4 |
| Radix liquiritiate | GC35 | 3'-Methoxyglabridin | 46.16 | 0.57 |
| Radix liquiritiate | GC36 | Glabrene | 46.27 | 0.44 |
| Radix liquiritiate | GC37 | (E)-3-[3,4-dihydroxy-5-(3-methylbut-2-enyl)phenyl]-1-(2,4-dihydroxyphenyl)prop-2-en-1-one | 46.27 | 0.31 |
| Radix liquiritiate | GC38 | quercetin | 46.43 | 0.28 |
| Radix liquiritiate | GC39 | Quercetin der. | 46.45 | 0.33 |
| Radix liquiritiate | GC40 | Calycosin | 47.75 | 0.24 |
| Radix liquiritiate | GC41 | 1,3-dihydroxy-9-methoxy-6-benzofurano[3,2-c]chromenone | 48.14 | 0.43 |
| Radix liquiritiate | GC42 | Semilicoisoflavone B | 48.78 | 0.55 |
| Radix liquiritiate | GC43 | Gancaonin B | 48.79 | 0.45 |
| Radix liquiritiate | GC44 | Medicarpin | 49.22 | 0.34 |
| Radix liquiritiate | GC45 | licochalcone G | 49.25 | 0.32 |
| Radix liquiritiate | GC46 | isorhamnetin | 49.6 | 0.31 |
| Radix liquiritiate | GC47 | Odoratin | 49.95 | 0.3 |
| Radix liquiritiate | GC48 | Gancaonin H | 50.1 | 0.78 |
| Radix liquiritiate | GC49 | kanzonols W | 50.48 | 0.52 |
| Radix liquiritiate | GC50 | Jaranol | 50.83 | 0.29 |
| Radix liquiritiate | GC51 | Gancaonin A | 51.08 | 0.4 |
| Radix liquiritiate | GC52 | Lupiwighteone | 51.64 | 0.37 |
| Radix liquiritiate | GC53 | licoisoflavanone | 52.47 | 0.54 |
| Radix liquiritiate | GC54 | Glabrone | 52.51 | 0.5 |
| Radix liquiritiate | GC55 | Glycyrin | 52.61 | 0.47 |
| Radix liquiritiate | GC56 | Glabranin | 52.9 | 0.31 |
| Radix liquiritiate | GC57 | Glabridin | 53.25 | 0.47 |
| Radix liquiritiate | GC58 | Xambioona | 54.85 | 0.87 |
| Radix liquiritiate | GC59 | Mairin | 55.38 | 0.78 |
| Radix liquiritiate | GC60 | Licoagroisoflavone | 57.28 | 0.49 |
| Radix liquiritiate | GC61 | 8-(6-hydroxy-2-benzofuranyl)-2,2-dimethyl-5-chromenol | 58.44 | 0.38 |
| Radix liquiritiate | GC62 | Licoagrocarpin | 58.81 | 0.58 |
| Radix liquiritiate | GC63 | naringenin | 59.29 | 0.21 |
| Radix liquiritiate | GC64 | 3-(2,4-dihydroxyphenyl)-8-(1,1-dimethylprop-2-enyl)-7-hydroxy-5-methoxy-coumarin | 59.62 | 0.43 |
| Radix liquiritiate | GC65 | (2S)-6-(2,4-dihydroxyphenyl)-2-(2-hydroxypropan-2-yl)-4-methoxy-2,3-dihydrofuro[3,2-g]chromen-7-one | 60.25 | 0.63 |
| Radix liquiritiate | GC66 | Gancaonin G | 60.44 | 0.39 |
| Radix liquiritiate | GC67 | Glyzaglabrin | 61.07 | 0.35 |
| Radix liquiritiate | GC68 | Glypallichalcone | 61.6 | 0.19 |
| Radix liquiritiate | GC69 | 1,3-dihydroxy-8,9-dimethoxy-6-benzofurano[3,2-c]chromenone | 62.9 | 0.53 |
| Radix liquiritiate | GC70 | Licoricone | 63.58 | 0.47 |
| Radix liquiritiate | GC71 | glyasperin B | 65.22 | 0.44 |
| Radix liquiritiate | GC72 | liquiritin | 65.69 | 0.74 |
| Radix liquiritiate | GC73 | 3-(3,4-dihydroxyphenyl)-5,7-dihydroxy-8-(3-methylbut-2-enyl)chromone | 66.37 | 0.41 |
| Radix liquiritiate | GC74 | formononetin | 69.67 | 0.21 |
| Radix liquiritiate | GC75 | 1-Methoxyphaseollidin | 69.98 | 0.64 |
| Radix liquiritiate | GC76 | (2R)-7-hydroxy-2-(4-hydroxyphenyl)chroman-4-one | 71.12 | 0.18 |
| Radix liquiritiate | GC77 | Vestitol | 74.66 | 0.21 |
| Radix liquiritiate | GC78 | Inermine | 75.18 | 0.54 |
| Radix liquiritiate | GC79 | glyasperin F | 75.84 | 0.54 |
| Radix liquiritiate | GC80 | Licochalcone B | 76.76 | 0.19 |
| Radix liquiritiate | GC81 | Phaseol | 78.77 | 0.58 |
| Radix liquiritiate | GC82 | shinpterocarpin | 80.3 | 0.73 |
| Radix liquiritiate | GC83 | licopyranocoumarin | 80.36 | 0.65 |
| Radix liquiritiate | GC84 | 7,2',4'-trihydroxy－5-methoxy-3－arylcoumarin | 83.71 | 0.27 |
| Radix liquiritiate | GC85 | Glycyrol | 90.78 | 0.67 |

Table 8 Basic information on the ingredients of Radix liquiritiae (GC)

| **Traditional Chinese Medicine** | **Identifier** | **Full name** | **OB(%)** | **DL** |
| --- | --- | --- | --- | --- |
| Artemisia adamsii Bess | ASH1 | DMQT | 42.6 | 0.37 |
| Artemisia adamsii Bess | ASH2 | [(2S)-2-[[(2S)-2-(benzoylamino)-3-phenylpropanoyl]amino]-3-phenylpropyl] acetate | 58.02 | 0.52 |
| Artemisia adamsii Bess | ASH3 | EUPATIN | 50.8 | 0.41 |
| Artemisia adamsii Bess | ASH4 | isorhamnetin | 49.6 | 0.31 |
| Artemisia adamsii Bess | ASH5 | Tamarixetin | 32.86 | 0.31 |
| Artemisia adamsii Bess | ASH6 | kaempferol | 41.88 | 0.24 |
| Artemisia adamsii Bess | ASH7 | Stigmasterol | 43.83 | 0.76 |
| Artemisia adamsii Bess | ASH8 | luteolin | 36.16 | 0.25 |

Table 9 Basic information on the ingredients of Artemisia adamsii Bess (ASH)

| **Traditional Chinese Medicine** | **Identifier** | **Full name** | **OB(%)** | **DL** |
| --- | --- | --- | --- | --- |
| Radix Inulae | ZMX1 | lappadilactone | 38.56 | 0.73 |
| Radix Inulae | ZMX2 | Mairin | 55.38 | 0.78 |
| Radix Inulae | ZMX3 | sitosterol | 36.91 | 0.75 |
| Radix Inulae | ZMX4 | Stigmasterol | 43.83 | 0.76 |

Table 10 Basic information on the ingredients of Radix Inulae (ZMX)

| **Traditional Chinese Medicine** | **Identifier** | **Full name** | **OB(%)** | **DL** |
| --- | --- | --- | --- | --- |
| Paeonia Lactiflora | BS1 | sitosterol | 36.91 | 0.75 |
| Paeonia Lactiflora | BS2 | beta-sitosterol | 36.91 | 0.75 |
| Paeonia Lactiflora | BS3 | kaempferol | 41.88 | 0.24 |
| Paeonia Lactiflora | BS4 | (3S,5R,8R,9R,10S,14S)-3,17-dihydroxy-4,4,8,10,14-pentamethyl-2,3,5,6,7,9-hexahydro-1H-cyclopenta[a]phenanthrene-15,16-dione | 43.56 | 0.53 |
| Paeonia Lactiflora | BS5 | Lactiflorin | 49.12 | 0.8 |
| Paeonia Lactiflora | BS6 | paeoniflorin | 53.87 | 0.79 |
| Paeonia Lactiflora | BS7 | Mairin | 55.38 | 0.78 |

Table 11 Basic information on the ingredients of Paeonia Lactiflora (BS)

| **Traditional Chinese Medicine** | **Identifier** | **Full name** | **GI Absorption** | **Druglikeness** |
| --- | --- | --- | --- | --- |
| Anisodamine | SLD1 | apoatropine | High | Yes |
| Anisodamine | SLD2 | n,n,n',n'-tetramethyl-holarrhimine | High | Yes |
| Anisodamine | SLD3 | tetramethyl diaminobutane | High | Yes |
| Anisodamine | SLD4 | tropine | High | Yes |
| Anisodamine | SLD5 | tropinone | High | Yes |

Table 12 Basic information on the ingredients of Anisodamine (SLD)

| **Traditional Chinese Medicine** | **Identifier** | **Full name** | **GI Absorption** | **Druglikeness** |
| --- | --- | --- | --- | --- |
| dandelion | PGY1 | 1,6,6-trimethyl-7-(3-oxobut-1-enyl)-3,8-dioxatricyclo[5.1.0.0(2,4)]octan-5-one | High | Yes |
| dandelion | PGY2 | 2-T-butyl-5-methyl-[1,3]dioxolane-4-carboxylic acid | High | Yes |
| dandelion | PGY3 | 3-methoxy-4-hydroxybenzaldehyd | High | Yes |
| dandelion | PGY4 | 4-hydroxy-4-methyl-2-pentanone | High | Yes |
| dandelion | PGY5 | 5,10-diethoxy-2,3,7,8-tetrahydro-1H,6H-dipyrrolo[1,2-A.1',2'-D]pyrazine | High | Yes |
| dandelion | PGY6 | 6-acetyl-4,4,7-trimethylbicyclo[4.1.0]heptan-2-one | High | Yes |
| dandelion | PGY7 | arsanin | High | Yes |
| dandelion | PGY8 | artecalin | High | Yes |
| dandelion | PGY9 | benzenecarboxylic acid | High | Yes |
| dandelion | PGY10 | caffeic acid | High | Yes |
| dandelion | PGY11 | chlorogenic acid | High | Yes |
| dandelion | PGY12 | desacetylmatricarin | High | Yes |
| dandelion | PGY13 | esculetin | High | Yes |
| dandelion | PGY14 | Ethyl caffeate | High | Yes |
| dandelion | PGY15 | ethyl p-hydroxyphenylacetate | High | Yes |
| dandelion | PGY16 | linolenic acid | High | Yes |
| dandelion | PGY17 | Methyl caffeate | High | Yes |
| dandelion | PGY18 | myristic acid | High | Yes |
| dandelion | PGY19 | palmitic acid | High | Yes |
| dandelion | PGY20 | phenylacetic acid | High | Yes |
| dandelion | PGY21 | p-hydroxybenzoate | High | Yes |
| dandelion | PGY22 | p-hydroxyphenylpropionic acid | High | Yes |
| dandelion | PGY23 | protocatechuic aldehyde | High | Yes |
| dandelion | PGY24 | quercetin | High | Yes |
| dandelion | PGY25 | scopoletin | High | Yes |
| dandelion | PGY26 | (+)-Syringaresinol | High | Yes |
| dandelion | PGY27 | taraxicin | High | Yes |
| dandelion | PGY28 | taraxinic acid | High | Yes |
| dandelion | PGY29 | trans-p-coumaryl alcohol | High | Yes |
| dandelion | PGY30 | trans-p-coumaryl aldehyde | High | Yes |

Table 13 Basic information on the ingredients of dandelion (PGY)

| **Traditional Chinese Medicine** | **Identifier** | **Full name** | **OB(%)** | **DL** |
| --- | --- | --- | --- | --- |
| Magnolia officinalis | HP1 | Eucalyptol | 60.62 | 0.32 |
| Magnolia officinalis | HP2 | Neohesperidin | 57.44 | 0.27 |

Table 14 Basic information on the ingredients of Magnolia officinalis (HP)

| **Traditional Chinese Medicine** | **Identifier** | **Full name** | **OB(%)** | **DL** |
| --- | --- | --- | --- | --- |
| Kudzu root | GG1 | beta-sitosterol | 36.91 | 0.75 |
| Kudzu root | GG2 | Daidzein-4,7-diglucoside | 47.27 | 0.67 |
| Kudzu root | GG3 | 3'-Methoxydaidzein | 48.57 | 0.24 |
| Kudzu root | GG4 | formononetin | 69.67 | 0.21 |

Table 15 Basic information on the ingredients of Kudzu root (GG)

| **Compared Samples** | **Num. of Total Sig.** | **Num. of Sig.Up** | **Num. of Sig.down** |
| --- | --- | --- | --- |
| XDZ1_vs_XAZ1.neg | 4,328 | 2,728 | 1,600 |
| XDZ1_vs_XAZ1.pos | 4,864 | 2,866 | 1,998 |
| XDZ1_vs_XBZ1.neg | 4,335 | 2,717 | 1,618 |
| XDZ1_vs_XBZ1.pos | 4,881 | 2,864 | 2,017 |
| XDZ1_vs_XCZ1.neg | 4,334 | 2,737 | 1,597 |
| XDZ1_vs_XCZ1.pos | 4,868 | 2,875 | 1,993 |
| XDZ1_vs_XAZ1.merge | 9,225 | 5,610 | 3,615 |
| XDZ1_vs_XBZ1.merge | 9,257 | 5,598 | 3,659 |
| XDZ1_vs_XCZ1.merge | 9,249 | 5,634 | 3,615 |
| XDZ2_vs_XAZ2.neg | 4,420 | 2,707 | 1,713 |
| XDZ2_vs_XAZ2.pos | 4,975 | 2,870 | 2,105 |
| XDZ2_vs_XBZ2.neg | 4,411 | 2,718 | 1,693 |
| XDZ2_vs_XBZ2.pos | 4,948 | 2,830 | 2,118 |
| XDZ2_vs_XCZ2.neg | 4,409 | 2,689 | 1,720 |
| XDZ2_vs_XCZ2.pos | 4,948 | 2,842 | 2,106 |
| XDZ2_vs_XAZ2.merge | 9,384 | 5,575 | 3,809 |
| XDZ2_vs_XBZ2.merge | 9,377 | 5,570 | 3,807 |
| XDZ2_vs_XCZ2.merge | 9,375 | 5,563 | 3,812 |

Table 16 Different Metabolite Screening

Compared Samples: Compare sample pairs, A_VS_ B represents B for differential analysis compared to A;

Num of Total Sig: The total number of metabolites with significant differences;

Num of Sig Up: The total number of significantly upregulated metabolites;

Num of Sig down: The total number of significantly downregulated metabolites.
